# *Salvia miltiorrhiza* and the Volatile of *Dalbergia odorifera* Attenuate Chronic Myocardial Ischemia Injury in a Pig Model: A Metabonomic Approach for the Mechanism Study

**DOI:** 10.1155/2021/8840896

**Published:** 2021-04-28

**Authors:** Rui Lin, Fei Mu, Yao Li, Jialin Duan, Meina Zhao, Yue Guan, Kedi Liu, Yang Bai, Aidong Wen, Peifeng Wei, Jingwen Wang, Miaomiao Xi

**Affiliations:** ^1^Department of Pharmacy, Xijing Hospital, Fourth Military Medical University, Xi'an, Shaanxi 710032, China; ^2^Northwest University, Faculty of Life Science & Medicine, Key Laboratory Resource Biology & Biotechnology in Western China, Ministry of Education, Xi'an, Shaanxi 710069, China; ^3^College of Pharmacy, Shaanxi University of Chinese Medicine, Xianyang, Shaanxi 712046, China; ^4^National Drug Clinical Trial Institute, The Second Affiliated Hospital, Shaanxi University of Chinese Medicine, Xianyang 712000, China; ^5^TANK Medicinal Biology Institute of Xi'an, Xi'an, Shaanxi 710032, China

## Abstract

*Salvia miltiorrhiza* (SM) coupled with *Dalbergia odorifera* (DO) has been used to relieve cardiovascular diseases in China for many years. Our previous studies have integrated that SM—the volatile oil of DO (SM-DOO)—has a cardioprotective effect on chronic myocardial ischemia based on a pharmacological method, but the cardioprotective mechanism has not been elucidated completely in the metabonomic method. In the present study, a metabonomic method based on high-performance liquid chromatography time-of-flight mass spectrometry (HPLC-Q-TOF-MS) was performed to evaluate the effects of SM-DOO on chronic myocardial ischemia induced by an ameroid constrictor, which was placed on the left anterior descending coronary artery (LAD) of pigs. Pigs were divided into three groups: sham, model, and SM-DOO group. With multivariate analysis, a clear cluster among the different groups was obtained and the potential biomarkers were recognized. These biomarkers were mainly related to energy metabolism, glucose metabolism, and fatty acid metabolism. Furthermore, the protein expressions of phosphorylated AMP-activated protein kinase (p-AMPK) and glucose transporter-4 (GLUT4) were significantly upregulated by SM-DOO. The result indicated that SM-DOO could regulate the above biomarkers and metabolic pathways, especially energy metabolism and glucose metabolism. By analyzing and verifying the biomarkers and metabolic pathways, further understanding of the cardioprotective effect of SM-DOO with its mechanism was evaluated. Metabonomic is a reliable system biology approach for understanding the cardioprotective effects of SM-DOO on chronic myocardial ischemia and elucidating the mechanism underlying this protective effect.

## 1. Introduction

Coronary artery disease is the largest composition of cardiovascular diseases, which is the leading cause of morbidity and mortality [1, 2]. Coronary artery disease is a threat to human health and safety but also brings heavy economic and social costs [3]. Previous investigations suggested beneficial effects of traditional Chinese medicine in the treatment of coronary artery disease, including *Salvia miltiorrhiza* (SM), *Panax notoginseng*, *Ligusticum chuanxiong*, and *Dalbergia odorifera* (DO) [4–6]. SM possesses wide-ranging pharmacological effects, such as anti-inflammatory, antioxidant, antitumor, antiatherosclerosis, and antidiabetic effects [7]. DO has a variety of pharmacological activities, including anti-inflammatory, antiangina, antioxidative, and other activities [8]. The combination of SM and DO can effectively treat cardiovascular diseases, such as GuanXin II prescription, QiShenYiQi drop pill, Guanxin Danshen formula, and Xiangdan injection [9–12]. Previously, we reported that SM—the volatile oil of DO (SM-DOO)—exhibited a significant effect in myocardial ischemia/reperfusion (MI/R) injury rats in both traditional pharmacological methods and metabonomic methods [13, 14]. On this basis of these data, traditional pharmacological methods (myocardial injury markers, myocardial oxygen consumption, ventricular function, and myocardial tissue damage) were used to evaluate the therapeutic effect of SM-DOO on chronic myocardial ischemia in pigs [15]. Results showed that the chronic myocardial model was successfully constructed and the SM-DOO treatment was effective. However, a more systematic approach to elucidating its possible mechanisms is lacking.

Metabonomic is widely used for the drug efficacy and toxicology, disease diagnosis, and biomarker identification [16]. Metabonomic analysis can detect physiological or pathological changes by identifying small molecular metabolites in samples of biological systems (including plasma, urine, feces, saliva, and tissues) to identify clinical biomarkers and therapeutic targets [17, 18]. In recent years, among the most recently emerged omics fields, metabonomic has been more widely used to reveal the mechanism of cardiovascular health and disease [19]. A range of technical approaches have been used for analysis of metabolites in biospecimens, including nuclear magnetic resonance (NMR) and mass spectrometry (MS). Metabolite profiling by liquid chromatography-mass spectrometry (LC-MS) has been considered to be one of the most commonly used techniques in high sensitivity, selectivity, and reproducibility in data acquisition biomarker discovery [20, 21].

Energy metabolism imbalance is an important characteristic of cardiovascular diseases. AMP-activated protein kinase (AMPK) is a serine-threonine kinase, which functions as a cardiac energy sensor and maintains energy homeostasis. Research suggests that AMPK activation can regulate glucose and fatty acid metabolism, mitochondrial function, apoptosis, etc. [22]. During ischemia, activated AMPK can translocate glucose transporter-4 (GLUT4) to the sarcolemma and then increase the uptake of glucose and regulate glucose metabolism [23, 24]. Hence, AMPK/GLUT4 may be a potential pathway for the treatment of cardiovascular diseases.

With the application of high-performance liquid chromatography time-of-flight mass spectrometry (HPLC-Q-TOF-MS) analysis, the purpose of this study was to identify plasma biomarker metabolites associated with chronic myocardial ischemia and illustrate the possible underlying mechanism of SM-DOO against chronic myocardial ischemia. Additionally, the protein expressions of AMPK and GLUT4 were detected to evaluate whether the energy metabolism and glucose metabolism serve as the potential metabolism pathway for the intervention or treatment of chronic myocardial ischemia.

## 2. Materials and Methods

### 2.1. Chemical and Reagents

Deionized water was prepared by using the Milli-Q water purification system (EMD Millipore, Bedford, MA, USA). HPLC-grade acetonitrile was supplied by Thermo Fisher Scientific Inc. (Waltham, MA, USA). MS-grade formic acid was purchased from Thermo Fisher scientific Inc. (Waltham, MA, USA). All other reagents and solvents were of analytical grade. The extract of *Salvia miltiorrhiza* (SM) was purchased from Xi'an Honson Biotechnology Co., Ltd. (Xi'an, China, batch number 161025). The volatile oil of *Dalbergia odorifera* (DOO) was purchased from Jishui Natural Perfume Oil Technology Co., Ltd. (Jiangxi, China, batch number XC20160918). The assay kit for glucose (Glu), lactic acid (LD), nonesterified fatty acid (NEFA), caspase-3, ATP, and ATPase was purchased from Nanjing Jiancheng Bioengineering Institute (Nanjing, China). The antibodies, including AMPK, p-AMPK, *β*-actin, and GLUT4, were purchased from Cell Signaling Technologies, Inc. (Danvers, MA, USA). The secondary antibody (anti-rabbit IgG-B; cat. no. sc-53804) was purchased from Santa Cruz Biotechnology, Inc. (Dallas, TX, USA).

### 2.2. Herbal Extraction

SM was dried at a temperature of 50°C and pulverized into powder (<1 mm). Then, 150 g SM was soaked in 8-fold the volume of water for 30 min at room temperature and extracted for 3 h (1.5 h/reflux), followed by reflux extraction with 75% ethanol. The suspension was filtered, concentrated under reduced pressure, and freeze-dried. The DOO was isolated by steam distillation for 5 h with a yield of 0.5% DOO and then stored at 4°C.

### 2.3. Animal Treatment

18 male pigs (25-30 kg) were provided by the Institute of Experiment Animals in the Fuwai Hospital Experimental Animal Center (permission number of 0072-1-27-HX (F). They were kept under standardized living temperature (25 ± 1°C) and relative humidity (55 ± 5%). After 1-week-long adaptation period, all the pigs were separated into three groups with six animals each group at random: sham group (oral administration of regular chow and sham-operated without ameroid constrictor implantation), model group (oral administration of regular chow), and SM-DOO group (oral administration of regular chow with SM 1 g/kg/day-DOO 0.1 ml/kg/day). Pigs were fed twice a day and water supplied free.

Following 4 weeks of dietary modification, all animals were anesthetized with pentobarbital sodium (25 mg/kg), intubated, and maintained with a gas mixture of oxygen at 1.5-2 l/min and 3% isoflurane. Pigs were on right lateral decubitus, and left thoracotomy was then performed in the 3 to 4th intercostal space. Heparin (80 IU/kg) was administered to help prevent thrombin formation. An ameroid constrictor (Research Instruments NW, Inc., Lebanon, OR, USA) (approximate size: 2.5 mm) was placed on the left anterior descending coronary artery (LAD). Sham group pigs underwent surgery, but the ameroid constriction was not placed around the LAD. After surgery, penicillin (6,400,000 U/day, intramuscularly) was administered to all pigs to protect against infection. All experimental procedures on pigs were carried out under the approval of the Animal Ethics Committee of Fuwai Hospital and were strictly in accordance with the relevant ethics regulations. After an 8-week follow-up, blood samples and heart tissues were collected for future metabonomic study.

### 2.4. Biochemical Analysis

A blood sample was collected and allowed to clot 30 min and then centrifuged at 3500 rpm at 4°C for 10 min. The levels of NEFA, LD, and Glu were measured using assay kits or a pig specific enzyme-linked immunosorbent assay (ELISA) kit according to the manufacturer's protocols. Left ventricle homogenates were centrifuged at 10000 rpm at 4°C for 10 min. The supernatant was used for testing the content of ATP, Na^+^K^+^-ATPase, Ca^++^-ATPase, and Ca^++^Mg^++^-ATPase. Then, the activity of caspase-3 was measured according to the manufacturer's protocols.

### 2.5. Sample Collection and Preparation

At the end of the experiment (8th week), all pigs were euthanatized with potassium chloride (2 mmol/kg), and blood samples were collected into tubes without shaking. Then, centrifugation was performed at 3500 rpm for 10 min, and the supernatant was subsequently centrifuged at 10000 rpm for 5 min at 4°C. The resultant plasma samples were pipetted into tubes and stored at -80°C until analysis.

Prior to analysis, the plasma samples were thawed at ambient temperature. 1500 *μ*l of acetonitrile was added into 500 *μ*l of plasma, vortexed for 60 seconds, and centrifuged (12000 rpm) for 10 min to obtain suspension. Repeated precipitation of protein was conducted 3 times. The supernatants for plasma were transferred and evaporated to dryness under a gentle stream of nitrogen. The residue was reconstituted in 500 *μ*l of acetonitrile-water containing 0.2% formic acid (*v*/*v*, 70 : 30) and was filtered through 0.22 *μ*m membrane for HPLC-Q-TOF-MS analysis.

### 2.6. Chromatographic Analysis

The pigs' plasma was evaluated using an Agilent1200 LC system equipped with a G6520 Accurate Mass Quadrupole Time-of-Flight mass spectrometer system (Agilent Technologies, USA). Chromatographic separation was performed on an Agilent TC-C18 column (150 mm × 4.6 mm, 5 *μ*m). The analytical column temperature was maintained at 30°C, and the flow rate was 0.8 ml/min. The mobile phase consisted of solution A (0.2% formic acid in water) and solution B (acetonitrile). The gradient elution was optimized as follows: 0-15 min, 10-30% B; 15-40 min, 30-32% B; 40-85 min, 32-55% B; 85-100 min, 55-80% B; and 100-120 min, 100% B.

The capillary voltages were set at 3500 V in a negative mode and 4000 V in a positive mode, respectively. Other parameters were as follows: gas temperature of 350°C, drying gas of 9 l/min, nebulizer of 35 psig, fragmentor voltage of 135 V, and skimmer voltage of 65 V. Data were collected in a centroid mode from 100 to 1100 m/z. The biomarker candidates were further analyzed by MS/MS with the collision energies 10, 20, 30, and 40 eV.

To obtain adequate information of the metabolites, both positive and negative ion modes of HPLC-Q-TOF-MS were applied to analyze the plasma samples of pigs.

### 2.7. Data Analysis and Pattern Recognition

The HPLC-Q-TOF-MS raw data firstly transformed into mzXML data file format and then processed by XCMS. After the data preprocessing, data was imported into the SIMCA 14.1 (Umetrics AB, Umea, Sweden) to perform principal component analysis (PCA), partial least squares discriminant analysis (PLS-DA), and orthogonal partial least squares discriminant analysis (OPLS-DA). A permutation test (200 iterations) was performed for validating and overfitting the OPLS-DA model. Plasma potential metabolites among the groups were selected according to the variable importance for projection (VIP > 1) and *P* value of analysis of variance (ANOVA, *P* < 0.05) in this study. The databases HMDB (http://www.hmdb.ca), METLIN (https://metlin.scripps.edu), KEGG (http://www.kegg.jp), etc. were used to identify the potential metabolites. Then, the potential metabolic pathways were analyzed by using MetaboAnalyst 4.0.

### 2.8. Western Blotting Analysis

Proteins were extracted from myocardial tissue, and the concentration of the lysates was determined according to the bicinchoninic acid (BCA) method using the Protein Quantitative Analysis Kit (Jiancheng, Nanjing, China). Fractionation was performed by 10% sodium dodecyl sulfate-polyacrylamide gel electrophoresis. After separation, the proteins were transferred onto polyvinylidene difluoride membranes, blocked for 1 h at 37°C with 5% nonfat dried milk, washed 3 times with Tween-Tris-buffered saline, and incubated with primary antibodies (AMPK, p-AMPK, *β*-actin, and GLUT4) diluted to 1 : 1000 overnight at 4°C. After 3 times washing as described above, the membranes were incubated with secondary antibodies (1 : 5000) at 37°C. Then, immunoreactive bands were detected using the enhanced chemiluminescence method and semiquantifications using ImageJ software (NIH, Bethesda, MD, USA).

### 2.9. Statistical Analysis

Data was expressed as the mean ± standard deviation (SD). One-way ANOVA followed by Tukey' test for multiple comparisons was employed to compare the means between groups. The statistical software used was GraphPad Prism (version 6.02, GraphPad Software Inc., La Jolla, CA, USA). The level of significance was set at *P* < 0.05.

## 3. Results

### 3.1. HPLC-Q-TOF-MS Profiling of Pigs' Plasma

**Figure 1:**
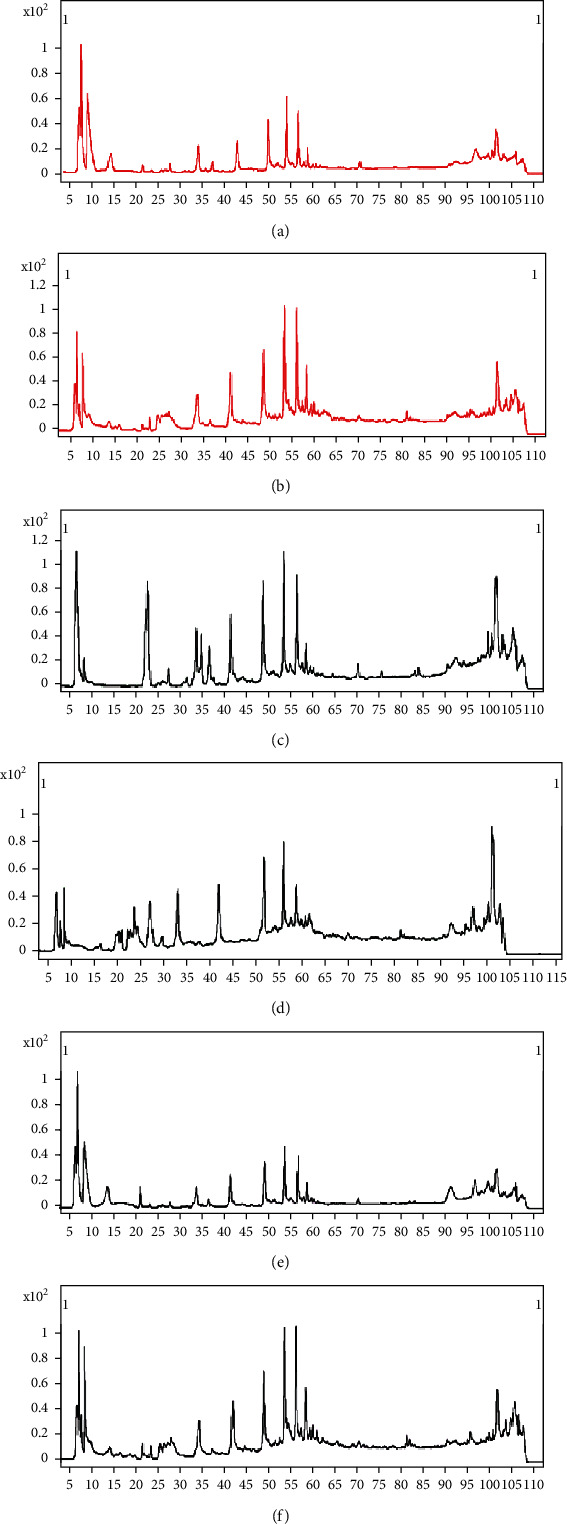
Typical example of TIC chromatogram of the pigs' plasma based on HPLC-Q-TOF-MS: (a) the TIC of the sham group in a negative mode; (b) the TIC of the sham group in a positive mode; (c) the TIC of the model group in a negative mode; (d) the TIC of the model group in a positive mode; (e) the TIC of the SM-DOO group in a negative mode; (f) the TIC of the SM-DOO group in a positive mode.

The metabolic profiles of plasma samples were acquired by HPLC-Q-TOF-MS in both negative and positive modes. Representative total ion chromatograms (TIC) of the sham group, model group, and SM-DOO group are shown in Figure 1. Compared with the model group, the TIC of the SM-DOO group was more similar to that of the sham group.

### 3.2. HPLC-Q-TOF-MS Metabolic Profiling of Plasma and Multivariate Data Analysis

**Figure 2:**
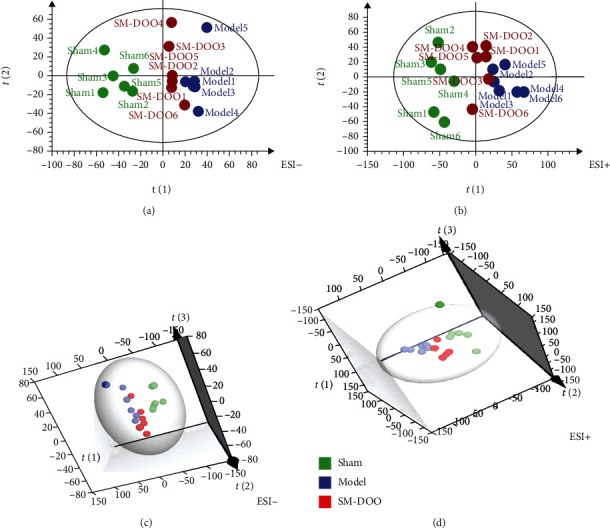
Score plots of plasma in each group based on HPLC-Q-TOF-MS in the negative and positive modes: (a) 2D PCA score plot in a negative mode; (b) 2D PCA score plot in a positive mode; (c) 3D PCA score plot in a negative mode; (d) 3D PCA score plot in a positive mode.

**Figure 3:**
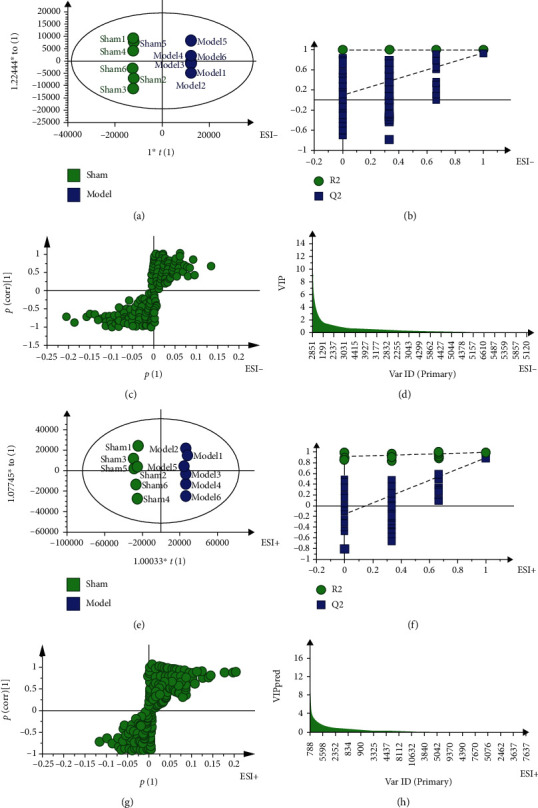
The metabolic profiles of OPLS-DA between the sham and model groups based on HPLC-Q-TOF-MS: (a) the score plot of OPLS-DA in a negative mode; (b) the corresponding validation plot based on 200 times permutation tests demonstrated the robustness of the OPLS-DA model in a negative mode; (c) the S-plot of OPLS-DA in a negative mode; (d) the VIP of OPLS-DA in a negative mode; (e) the score plot of OPLS-DA in a positive mode; (f) the corresponding validation plot based on 200 times permutation tests demonstrated the robustness of the OPLS-DA model in a positive mode; (g) the S-plot of OPLS-DA in a positive mode; (h) the VIP of OPLS-DA in a positive mode.

**Figure 4:**
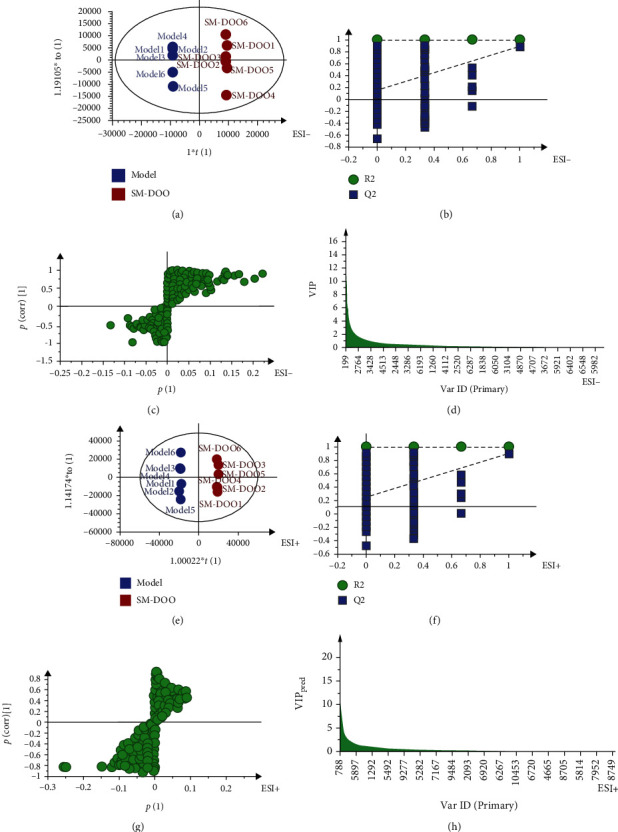
The metabolic profiles of OPLS-DA between the model and SM-DOO groups based on HPLC-Q-TOF-MS: (a) the score plot of OPLS-DA in a negative mode; (b) the corresponding validation plot based on 200 times permutation tests demonstrated the robustness of the OPLS-DA model in a negative mode; (c) the S-plot of OPLS-DA in a negative mode; (d) the VIP of OPLS-DA in a negative mode; (e) the score plot of OPLS-DA in a positive mode; (f) the corresponding validation plot based on 200 times permutation tests demonstrated the robustness of the OPLS-DA model in a positive mode; (g) the S-plot of OPLS-DA in a positive mode; (h) the VIP of OPLS-DA in a positive mode.

Both from PCA (Figure 2) and PLS-DA (in the supplementary file (available here)) results, the plasma of the sham group is distributed in the figure and well separated from the model and SM-DOO groups in both negative and positive modes. Moreover, compared with the model group, the SM-DOO group was much closer to the sham group. The results suggest that pretreatment of SM-DOO can restore the pathological process of chronic myocardial ischemia making some metabolites come back to the normal level. To search for the metabolic profiling changes of the groups, OPLS-DA was employed (Figures 3 and 4). As shown in Figures 3(a) and 3(e), there was an obvious separation between the sham group and model group, which suggested that the metabolites were changed in the model group. The parameters of the sham and model groups (negative mode: *R*^2^*Y* = 0.998, *Q*^2^ = 0.983; positive mode: *R*^2^*Y* = 0.997, *Q*^2^ = 0.89) were acceptable, which also indicated that the model was stably established. Additionally, there was distinguished classification metabolic profiling between the SM-DOO group and model group which are shown in Figures 4(a) and 4(e). The results of 200 times permutation tests indicated that all the established OPLS-DA models were credible, as the *R*^2^ and *Q*^2^ values were lower than the original ones (Figures 3(b), 3(f), 4(b), and 4(f)).

The S-plots (Figures 3(c), 3(g), 4(c), and 4(g)) and VIP plots (Figures 3(d), 3(h), 4(d), and 4(h)) were performed after the OPLS-DA model for selecting the potential biomarkers in both negative and positive modes. The farther away from the origin of the S-plot, the greater the contribution to potential biomarkers. The potential biomarkers were picked out according to the VIP > 1and*P* < 0.05.

### 3.3. Identification of Potential Biomarkers and Metabolic Pathway Analysis

Potential biomarkers were selected according to the VIP > 1 and *P* < 0.05 from the OPLS-DA between the sham and model groups. Finally, 13 metabolites were identified by searching databases such as HMDB (http://www.hmdb.ca/), KEGG (http://www.kegg.com/), and METLIN (http://metlin.scripps.edu/), including citric acid, malic acid, 2-oxoglutarate, glucose, glucose-6-phosphate, lactic acid, 3-hydroxybutyric acid, stearic acid, creatine, phenylalanine, L-alanine, glutamine, and L-isoleucine (Table 1).

**Figure 5:**
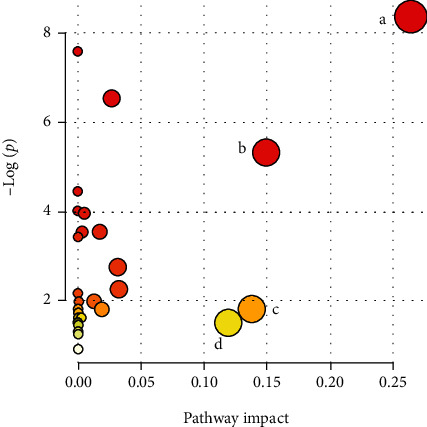
The topology analysis of the related metabolic pathway with MetaboAnalyst 4.0 (pathway impact ≥ 0.10): a: alanine, aspartate, and glutamate metabolism; b: citrate cycle (TCA cycle); c: pyruvate metabolism; and d: phenylalanine metabolism.

Based on the potential biomarkers of chronic myocardial ischemia pigs, the most relevant metabolic pathways were identified. According to the impact value (pathway impact ≥ 0.10), the metabolism pathways were recognized to be involved in chronic myocardial ischemia, including alanine, aspartate, and glutamate metabolism, citrate cycle (TCA cycle), pyruvate metabolism, and phenylalanine metabolism (Figure 5). These pathways might suggest potential as the targeted pathways of SM-DOO against chronic myocardial ischemia.

### 3.4. Analysis of Correlation between Biomarkers and Biochemical Indicators

**Figure 6:**
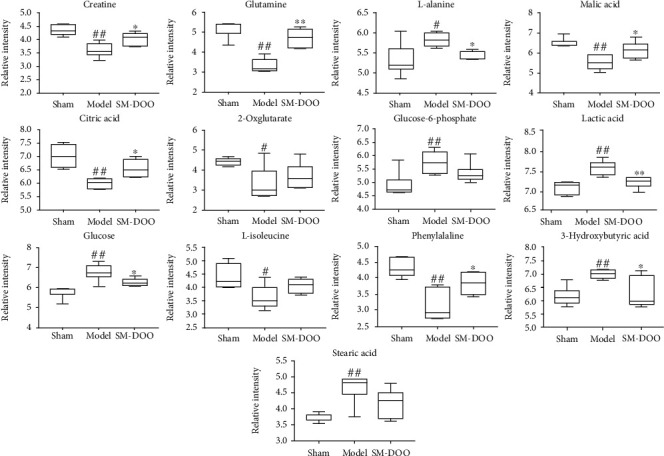
Box plots showed the normalized relative contents of these differential metabolites in different groups. All values are presented as the mean ± SD. ^#^*P* < 0.05, ^##^*P* < 0.01 vs. sham group; ^∗^*P* < 0.05, ^∗∗^*P* < 0.01 vs. model group.

**Figure 7:**
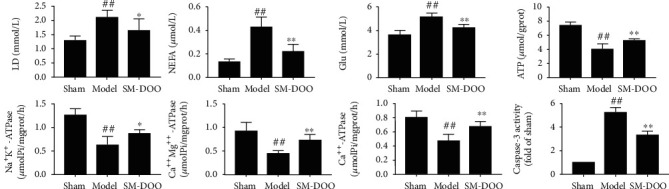
Changes of biochemical indicators of serum or myocardium in different groups. All values are presented as the mean ± SD. ^##^*P* < 0.01 vs. sham group; ^∗^*P* < 0.05, ^∗∗^*P* < 0.01 vs. model group.

Compared with the sham group, the levels of creatine, glutamine, malic acid, citric acid, 2-oxoglutarate, L-isoleucine, and phenylalanine were reduced, while L-alanine, glucose-6-phosphate, lactic acid, glucose, 3-hydroxybutyric acid, and stearic acid were advanced (Figure 6). After the administration of SM-DOO, the above indicators are adjusted to normal level. Biochemical indicators are shown in Figure 7, the levels of LD, NEFA, Glu, and caspase-3 were significantly elevated (*P* < 0.01) in the model group compared to the sham group, while the energy-related indicators such as ATP, Na^+^K^+^-ATPase, Ca^++^Mg^++^-ATPase, and Ca^++^-ATPase were significantly decreased (*P* < 0.01). After SM-DOO administration, the above indicators were significantly reversed (*P* < 0.05 or *P* < 0.01).

The Pearson correlation analysis was used to study the relationship between potential biomarkers and biochemical indicators (Table 2). Based on the correlation coefficient *r*, an absolute value greater than 0.7 is expressed as a strong correlation, indicating that creatine is strongly correlated with LD, NEFA, Glu, ATP, Ca^++^-ATPase, and caspase-3. Phenylalanine is strongly correlated with NEFA, Glu, ATP, Na^+^K^+^-ATPase, Ca^++^-ATPase, and caspase-3. Malic acid is strongly correlated with LD, NEFA, Ca^+^Mg^+^-ATPase, and caspase-3. Citric acid is strongly correlated with ATP, Na^+^K^+^-ATPase, Ca^++^-ATPase, and caspase-3. Moreover, L-alanine and Ca^+^Mg^+^-ATPase are strongly correlated. Lactic acid and LD, NEFA, Glu, Ca^++^-ATPase, and caspase-3 are strongly correlated. Glucose and NEFA, Glu, ATP, Ca^+^Mg^+^-ATPase, and caspase-3 are strongly correlated. These intrinsic relationship analyses may be significant for understanding the chronic myocardial ischemia mainly related to the energy metabolism and glucose metabolism.

### 3.5. Western Blot Validation

**Figure 8:**
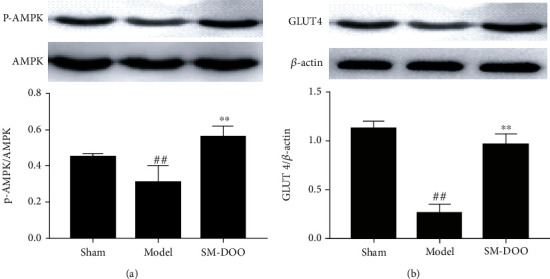
Effects of SM-DOO on the AMPK/GLUT4 pathway in heart tissues of pigs. p-AMPK, AMPK, GLUT4, and *β*-actin protein expressions were measured. The protein signals were quantitated by densitometry, and the graph shows their relative levels. All values are presented as the mean ± SD. ^##^*P* < 0.01 vs. sham group; ^∗∗^*P* < 0.01 vs. model group.

To validate the mechanism of SM-DOO protection against chronic myocardial ischemia, the expressions of AMPK and GLUT4 were assessed by Western blot (Figure 8). The result showed that the expression level of GLUT4 was decreased in the model group compared with the sham group. Compared with the model group, SM-DOO significantly increased the expression of GLUT4 (*P* < 0.01). There was no significant difference in the expression levels of AMPK between the groups. Compared with the model group, SM-DOO significantly upregulated the expression level of p-AMPK.

## 4. Discussion

Whether in developed or developing countries, cardiovascular disease health is still the focus of attention. Early diagnosis of cardiovascular disease is difficult but important, so there is a need for exploring rapid and useful biomarkers. Metabonomic is the comprehensive assessment of endogenous metabolites of a biological system in a holistic context. Metabonomic has suggestive advantages and efficacy in revealing the metabolic aspects of chronic myocardial ischemia.

In this study, HPLC-Q-TOF-MS metabonomic was used to screen the metabolic differences between normal and chronic myocardial ischemia in pigs and to explore the related metabolic pathways and the mechanism of chronic myocardial ischemia. After treatment of SM-DOO, the result showed that SM-DOO could have a significant therapeutic role in chronic myocardial ischemia and keep pigs near to the normal levels.

Representative TIC spectrums of HPLC-Q-TOF-MS were shown similar metabolites in three groups but also figure out some differences. Multivariate analysis was used to establish a classification model to distinguish between the sham and model groups and to identify potential diagnostic biomarkers for chronic myocardial ischemia. The results of analysis showed that citric acid, malic acid, 2-oxoglutarate, glucose, glucose-6-phosphate, lactic acid, 3-hydroxybutyric acid, stearic acid, creatine, phenylalanine, L-alanine, glutamine, and L-isoleucine were potential biomarkers. Otherwise, a box plot also helps us better understand the changes of metabolites among different groups. The metabolic pathways were identified by using MetaboAnalyst 4.0. According to the impact value (pathway impact ≥ 0.10), the metabolism pathways were recognized to be involved in chronic myocardial ischemia, including alanine, aspartate, and glutamate metabolism, TCA cycle, pyruvate metabolism, and phenylalanine metabolism.

**Figure 9:**
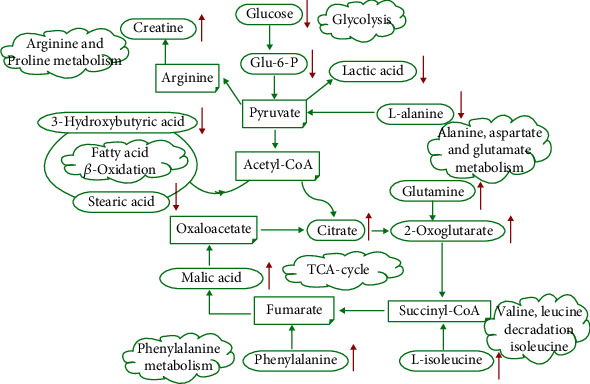
Potential metabolic pathways of SM-DOO treatment of chronic myocardial ischemia pigs induced by ameroid constrictor implantation. Glu-6-P: Glucose-6-phosphate; Acetyl-CoA: acetyl-coenzyme A; Succinyl-CoA: succinyl-coenzyme A.

To validate the findings in metabolic pathways, we also conducted correlation analysis using efficacy biochemical indicators and potential biomarkers. As shown in Table 2, we have confirmed that it may be significant for understanding the chronic myocardial ischemia mainly related to energy metabolism and glucose metabolism. Western blot showed that SM-DOO significantly upregulated the expression level of p-AMPK and GLUT4. According to the results, SM-DOO improved energy and glucose metabolism by activation of the AMPK and GLUT4 signaling pathways. The potential metabolic pathways of SM-DOO treatment of chronic myocardial ischemia pigs are shown in Figure 9.

### 4.1. Energy Metabolism

Creatine is an important indicator for maintaining energy homeostasis, protecting oxidative damage and improving health and survival [25, 26]. Creatine can increase creatine phosphate reserves, which can provide energy, protect mitochondria, and reduce cardiomyocyte apoptosis. In our study, a significant decrease in creatine was observed in the model group relative to the sham group, while in the SM-DOO group, the level reverts back nearly to normal level. This phenomenon showed that SM-DOO may regulate energy metabolism and reduce cardiomyocyte apoptosis, consistent with the changes of biochemical analysis (caspase-3 in Figure 7). Citric acid, malic acid, and 2-oxglutarate are intermediate products in the TCA cycle [27, 28]. Glutamine indirectly participates in the TCA cycle by 2-oxglutarate. The TCA cycle plays important roles in energy metabolism, and the decreased levels of citric acid, malic acid, 2-oxglutarate, and glutamine in the plasma of the model group suggested the disorder of energy metabolism. L-Isoleucine, as a TCA cycle intermediate, could be converted to succinyl-CoA which is an important step of the TCA cycle [29, 30]. Phenylalanine is a kind of essential amino acid and the precursor for tyrosine [31, 32]. In addition, tyrosine could degrade to the fumarate (TCA cycle intermediate) [33]. Therefore, the levels of L-isoleucine and phenylalanine were downregulated in the model group, which are indirect evidence of energy metabolism changes. Above all, those results showed that chronic myocardial ischemia downregulates energy metabolism, consistent with the decrease in the levels of energy-related indicators such as ATP, Na^+^K^+^-ATPase, Ca^++^Mg^++^-ATPase, and Ca^++^-ATPase in Figure 7. However, after treating with SM-DOO, the levels of citric acid, malic acid, 2-oxglutarate, L-isoleucine, and phenylalanine were restored to normal levels.

### 4.2. Glucose Metabolism

L-Alanine is an amino acid that is synthesized by the transamination of pyruvate and then released into the blood [34]. As one of the products of glycolysis, lactate has been shown to have an upward trend during myocardial ischemia [35]. In our study, we found that L-alanine and lactic acid level were significantly increased in chronic myocardial ischemia pigs than in normal pigs, consistent with the changes of biochemical analysis (LD in Figure 7). However, the levels of L-alanine and lactic acid in the SM-DOO group showed decrease. Glucose, as another energy substrate of the heart, produces pyruvate by glycolysis and then enters the TCA cycle [36]. Glucose-6-phosphate is produced from glucose and activities of glycolysis and TCA cycle. In the model group, the changes of metabolites of L-alanine, lactic acid, glucose, and glucose-6-phosphate might be linked to glucose metabolism changes. After administration of SM-DOO, the abnormalities of these metabolites were improved, which speculated that SM-DOO may ameliorate through glucose metabolism chronic myocardial ischemia.

### 4.3. Fatty Acid Metabolism

Fatty acids, especially *β*-oxidation of fatty acids, are the main energy substrates for the heart [37]. Furthermore, it has been reported that cardiac dysfunction altered fatty acid oxidation [38]. In this study, 3-hydroxybutyric acid and stearic acid were higher in the model group compared with the sham group and agree with the increase in the level of NEFA in Figure 7, which indicated that fatty acid metabolism was greatly disturbed when chronic myocardial ischemia occurred. Conversely, a decrease in 3-hydroxybutyric acid and stearic acid in the SM-DOO group in comparison to the model group was observed. The disorders of fatty acid metabolism were ameliorated by SM-DOO to protect the heart.

Our metabonomic results indicate effects of SM-DOO on energy metabolism, glucose metabolism, and fatty acid metabolism in pigs, especially in energy metabolism and glucose metabolism. It would help in understanding the mechanism of action of SM-DOO.

## 5. Conclusions

In the present study, the metabolic profiling of the pigs' plasma in chronic myocardial ischemia and the effect of SM-DOO were revealed using HPLC-Q-TOF-MS followed by multivariate analysis. The results confirmed that SM-DOO has cardioprotection effects on chronic myocardial ischemia pigs via regulation of metabolic pathways involved in energy metabolism, glucose metabolism, and fatty acid metabolism. Furthermore, the protein expression of research suggested that energy metabolism (upregulating the level of p-AMPK) and glucose metabolism (increasing the expression of GLUT4) may play as potential targets for the intervention or treatment of chronic myocardial ischemia. This study suggested that metabolic mechanism of SM-DOO for the treatment of chronic myocardial ischemia set the foundation for the diagnosis of chronic myocardial ischemia; also, the SM-DOO is widely used in a clinical setting.

## Figures and Tables

**Table 1:** Metabolites selected as biomarkers characterized in the plasma profile and their change trends.

Metabolites	ESI	*t*_R_ (min)	VIP	Model^a^		SM-DOO^b^		Related pathway
				*P*	Trend	*P*	Trend	
Creatine	(-)	4.44	7.16	0.0001	↓^∗^	0.0148	↑^∗^	Arginine and proline metabolism
Glutamine	(+)	59.23	7.43	0.0004	↓^∗^	0.001	↑^∗^	Alanine, aspartate, and glutamate metabolism
L-Alanine	(+)	32.17	11.59	0.049	↑^∗^	0.046	↓^∗^	Alanine, aspartate, and glutamate metabolism
Malic acid	(-)	6.56	1.36	0.0006	↓^∗^	0.0201	↑^∗^	Citrate cycle
Citric acid	(-)	70.98	2.95	0.0004	↓^∗^	0.0392	↑^∗^	Citrate cycle
2-Oxoglutarate	(-)	21.52	9.62	0.018	↓^∗^	0.5499	↑	Citrate cycle
Glucose-6-phosphate	(-)	33.14	14.86	0.0082	↑^∗^	0.2164	↓	Glycolysis
Lactic acid	(-)	52.98	1.27	0.0002	↑^∗^	0.003	↓^∗^	Glycolysis
Glucose	(-)	51.04	5.90	0.0002	↑^∗^	0.0396	↓^∗^	Glycolysis
L-Isoleucine	(+)	4.58	5.87	0.0109	↓^∗^	0.1643	↑	Valine, leucine, and isoleucine degradation
Unknown	(-)	43.16	3.05	0.0001	↓^∗^	0.0001	↑^∗^	Unknown
Phenylalanine	(+)	63.30	1.19	0.002	↓^∗^	0.0157	↑^∗^	Phenylalanine metabolism
3-Hydroxybutyric acid	(-)	9.29	7.12	0.0062	↑^∗^	0.0159	↓^∗^	*β*-Oxidation of fatty acid
Stearic acid	(-)	4.38	1.57	0.0016	↑^∗^	0.0956	↓	*β*-Oxidation of fatty acid

^a^Change trend compared with the sham group. ^b^Change trend compared with the model group. The levels of potential biomarkers were labeled with ↓ (downregulated) and ↑ (upregulated) (^∗^*P* < 0.05). ESI: electrospray ionization.

**Table 2:** Correlation analysis between efficacy biochemical indicators and potential biomarkers based on the Pearson correlation coefficient.

	LD	NEFA	Glu	ATP	Na^+^K^+^-ATPase	Ca^+^Mg^+^-ATPase	Ca^++^-ATPase	Caspase-3
Creatine	-0.722^∗∗^	-0.759^∗∗^	-0.734^∗∗^	0.725^∗∗^	0.623^∗∗^	0.581^∗^	0.715^∗∗^	-0.804^∗∗^
Glutamine	-0.524^∗^	-0.845^∗∗^	-0.750^∗∗^	0.754^∗∗^	0.819^∗∗^	0.666^∗∗^	0.714^∗∗^	-0.879^∗∗^
L-Alanine	0.527^∗^	0.616^∗∗^	0.674^∗∗^	-0.552^∗^	-0.500^∗^	-0.711^∗∗^	-0.419	0.663^∗∗^
Malic acid	-0.773^∗∗^	-0.792^∗∗^	-0.640^∗∗^	0.660^∗∗^	0.684^∗∗^	0.706^∗∗^	0.661^∗∗^	-0.732^∗∗^
Citric acid	-0.568^∗^	-0.594^∗∗^	-0.532^∗^	0.709^∗∗^	0.793^∗∗^	0.626^∗∗^	0.704^∗∗^	-0.780^∗∗^
2-Oxglutarate	-0.410	-0.530^∗^	-0.596^∗∗^	0.515^∗^	0.635^∗∗^	0.399	0.584^∗^	-0.635^∗∗^
Glucose-6-phosphate	0.436	0.578^∗^	0.550^∗^	-0.719^∗∗^	-0.582^∗^	-0.480^∗^	-0.571^∗^	0.669^∗∗^
Lactic acid	0.701^∗∗^	0.808^∗∗^	0.737^∗∗^	-0.620^∗∗^	-0.595^∗∗^	-0.699^∗∗^	-0.714^∗∗^	0.786^∗∗^
Glucose	0.626^∗∗^	0.777^∗∗^	0.703^∗∗^	-0.767^∗∗^	-0.661^∗∗^	-0.705^∗∗^	-0.671^∗∗^	0.753^∗∗^
L-Isoleucine	-0.427	-0.560^∗^	-0.560^∗^	0.513^∗^	0.667^∗∗^	0.401	0.764^∗∗^	-0.672^∗∗^
Phenylalanine	-0.608^∗∗^	-0.700^∗∗^	-0.827^∗∗^	0.736^∗∗^	0.773^∗∗^	0.642^∗∗^	0.716^∗∗^	-0.831^∗∗^
3-Hydroxybutyric acid	0.744^∗∗^	0.569^∗^	0.443	-0.571^∗^	-0.518^∗^	-0.546^∗^	-0.655^∗∗^	0.647^∗∗^
Stearic acid	0.514^∗^	0.644^∗∗^	0.617^∗∗^	-0.647^∗∗^	-0.526^∗^	-0.520^∗^	-0.719^∗∗^	0.734^∗∗^

^∗∗^Correlation is significant at the 0.01 level. ^∗^Correlation is significant at the 0.05 level.

## Data Availability

The data used to support the findings of this study are included within the article.

## Supplementary Materials

Supplementary Materials Score plots of plasma in each group based on HPLC-Q-TOF-MS in the negative and positive modes. (a) 2D PLS-DA score plot in a negative mode. (b) 2D PLS-DA score plot in a positive mode. (c) 3D PLS-DA score plot in a negative mode. (d) 3D PLS-DA score plot in a positive mode.
