# Corrigendum to “Salvia miltiorrhiza and the Volatile of Dalbergia odorifera Attenuate Chronic Myocardial Ischemia Injury in a Pig Model: A Metabonomic Approach for the Mechanism Study”

**DOI:** 10.1155/2022/9763253

**Published:** 2022-03-23

**Authors:** Rui Lin, Fei Mu, Yao Li, Jialin Duan, Meina Zhao, Yue Guan, Kedi Liu, Yang Bai, Aidong Wen, Peifeng Wei, Jingwen Wang, Miaomiao Xi

**Affiliations:** ^1^Department of Pharmacy, Xijing Hospital, Fourth Military Medical University, Xi'an, Shaanxi 710032, China; ^2^Northwest University, Faculty of Life Science & Medicine, Key Laboratory Resource Biology & Biotechnology in Western China, Ministry of Education, Xi'an, Shaanxi 710069, China; ^3^College of Pharmacy, Shaanxi University of Chinese Medicine, Xianyang, Shaanxi 712046, China; ^4^National Drug Clinical Trial Institute, The Second Affiliated Hospital, Shaanxi University of Chinese Medicine, Xianyang 712000, China; ^5^TANK Medicinal Biology Institute of Xi'an, Xi'an, Shaanxi 710032, China

In the article titled “Salvia miltiorrhiza and the Volatile of Dalbergia odorifera Attenuate Chronic Myocardial Ischemia Injury in a Pig Model: A Metabonomic Approach for the Mechanism Study” [1], the authors identified error in Figure 4, where (b) and (f) were mistakenly duplicated.  Figure 4(f) had been corrected as follows:

Additionally, the P-AMPK bands in Figure 8(a) and the GLUT-4 bands in Figure 8(b) were found to be duplicated. Figure 8(b) has been corrected as follows:

## Figures and Tables

**Figure 1:**
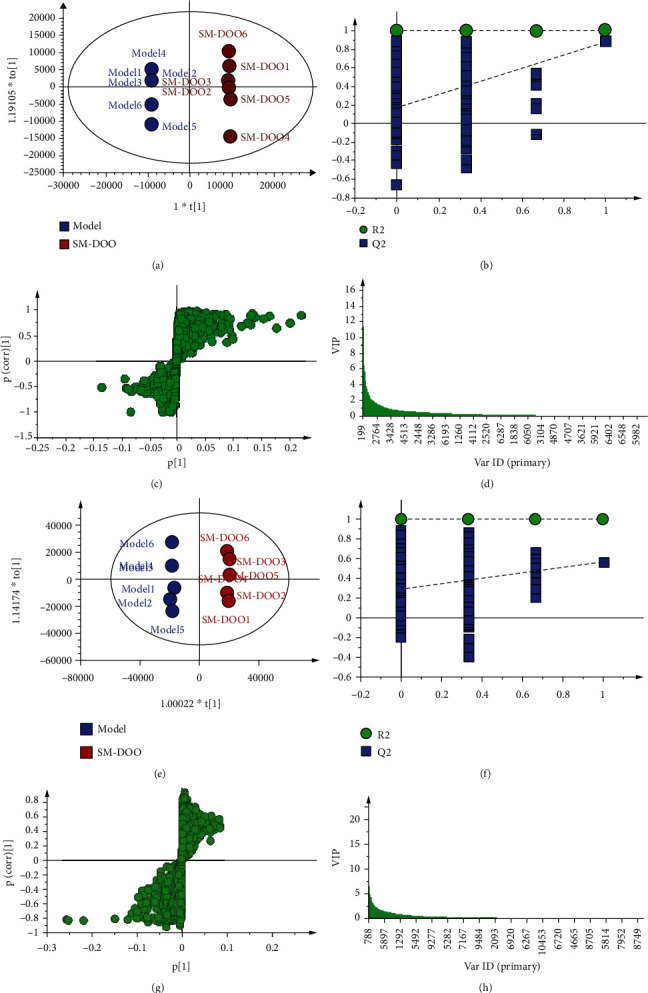
The metabolic profiles of OPLS-DA between the model and SM-DOO groups based on HPLC-Q-TOF-MS: (a) the score plot of OPLS-DA in a negative mode; (b) the corresponding validation plot based on 200 times permutation tests demonstrated the robustness of the OPLS-DA model in a negative mode; (c) the S-plot of OPLS-DA in a negative mode; (d) the VIP of OPLS-DA in a negative mode; (e) the score plot of OPLS-DA in a positive mode; (f) the corresponding validation plot based on 200 times permutation tests demonstrated the robustness of the OPLS-DA model in a positive mode; (g) the S-plot of OPLS-DA in a positive mode; (h) the VIP of OPLS-DA in a positive mode.

**Figure 2:**
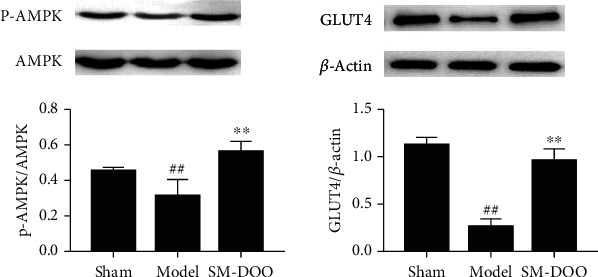
Effects of SM-DOO on the AMPK/GLUT4 pathway in heart tissues of pigs. p-AMPK, AMPK, GLUT4, and *β*-actin protein expressions were measured. The protein signals were quantitated by densitometry, and the graph shows their relative levels. All values are presented as the mean ± SD. ##*P* < 0*:*01 vs. sham group; ∗∗*P* < 0*:*01 vs. model group.
