# Supplementary material for: Salvia miltiorrhiza and the Volatile of Dalbergia odorifera Attenuate Chronic Myocardial Ischemia Injury in a Pig Model: A Metabonomic Approach for the Mechanism Study
Source: Oxid Med Cell Longev. 2021 Apr 28;2021:8840896. doi: 10.1155/2021/8840896 (PMC8099511; doi:10.1155/2021/8840896)
Supplement: Supplementary Materials — Score plots of plasma in each group based on HPLC-Q-TOF-MS in the negative and positive modes. (a) 2D PLS-DA score plot in a negative mode. (b) 2D PLS-DA score plot in a positive mode. (c) 3D PLS-DA score plot in a negative mode. (d) 3D PLS-DA score plot in a positive mode. [file 8840896.f1.pdf]

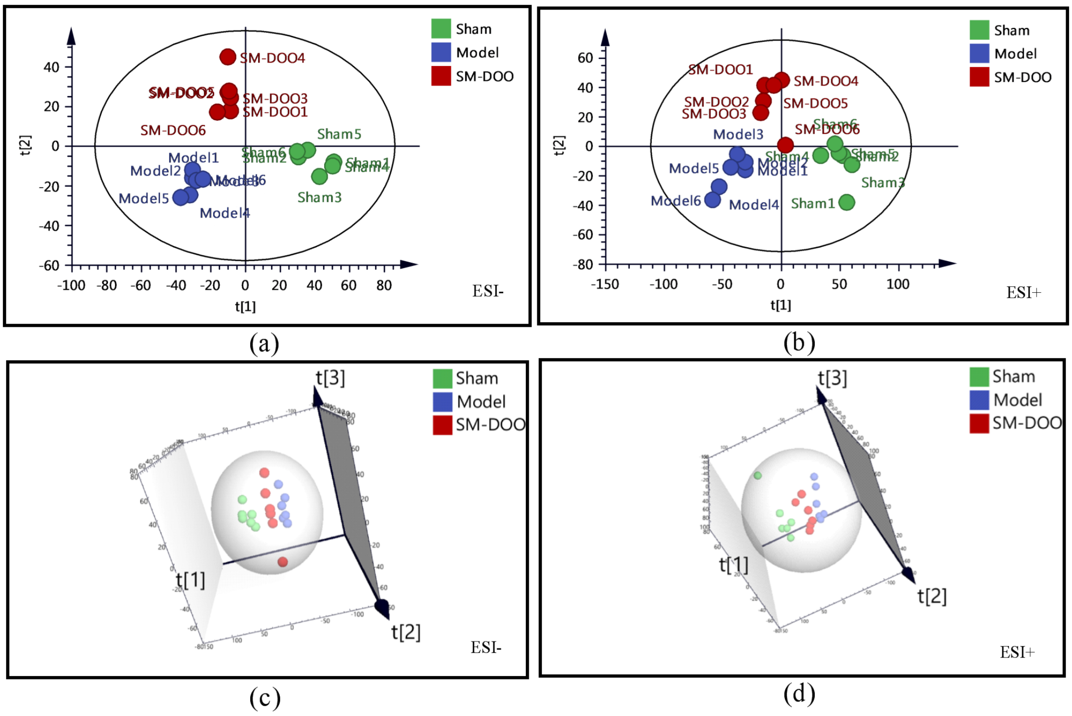

Score plots of plasma in each group based on HPLC-Q-TOF-MS in the negative and positive modes. (a)2D PLS-DA score plot in negative mode. (b) 2D PLS-DA score plot in positive mode. (c) 3D PLS-DA score plot in negative mode. (d) 3D PLS-DA score plot in positive mode.
